# Characteristics and Risk Factors for Intensive Care Unit Cardiac Arrest in Critically Ill Patients with COVID-19—A Retrospective Study

**DOI:** 10.3390/jcm10102195

**Published:** 2021-05-19

**Authors:** Kevin Roedl, Gerold Söffker, Dominic Wichmann, Olaf Boenisch, Geraldine de Heer, Christoph Burdelski, Daniel Frings, Barbara Sensen, Axel Nierhaus, Dirk Westermann, Stefan Kluge, Dominik Jarczak

**Affiliations:** 1 Department of Intensive Care Medicine, University Medical Centre Hamburg-Eppendorf, 20246 Hamburg, Germany; g.soeffker@uke.de (G.S.); d.wichmann@uke.de (D.W.); o.boenisch@uke.de (O.B.); deheer@uke.de (G.d.H.); c.burdelski@uke.de (C.B.); d.frings@uke.de (D.F.); b.sensen@uke.de (B.S.); nierhaus@uke.de (A.N.); s.kluge@uke.de (S.K.); d.jarczak@uke.de (D.J.); 2 Department of Interventional and General Cardiology, University Heart Centre Hamburg, 20246 Hamburg, Germany; d.westermann@uke.de

**Keywords:** COVID-19, coronavirus disease, multiple organ failure, intensive care unit, SARS-CoV-2, cardiac arrest, cardiopulmonary resuscitation, in-hospital cardiac arrest, ICU-CA

## Abstract

The severe acute respiratory syndrome coronavirus-2 (SARS-CoV-2) causing the coronavirus disease 2019 (COVID-19) led to an ongoing pandemic with a surge of critically ill patients. Very little is known about the occurrence and characteristic of cardiac arrest in critically ill patients with COVID-19 treated at the intensive care unit (ICU). The aim was to investigate the incidence and outcome of intensive care unit cardiac arrest (ICU-CA) in critically ill patients with COVID-19. This was a retrospective analysis of prospectively recorded data of all consecutive adult patients with COVID-19 admitted (27 February 2020–14 January 2021) at the University Medical Centre Hamburg-Eppendorf (Germany). Of 183 critically ill patients with COVID-19, 18% (*n* = 33) had ICU-CA. The median age of the study population was 63 (55–73) years and 66% (*n* = 120) were male. Demographic characteristics and comorbidities did not differ significantly between patients with and without ICU-CA. Simplified Acute Physiological Score II (SAPS II) (ICU-CA: median 44 points vs. no ICU-CA: 39 points) and Sequential Organ Failure Assessment (SOFA) score (median 12 points vs. 7 points) on admission were significantly higher in patients with ICU-CA. Acute respiratory distress syndrome (ARDS) was present in 91% (*n* = 30) with and in 63% (*n* = 94) without ICU-CA (*p* = 0.002). Mechanical ventilation was more common in patients with ICU-CA (97% vs. 67%). The median stay in ICU before CA was 6 (1–17) days. A total of 33% (*n* = 11) of ICU-CAs occurred during the first 24 h of ICU stay. The initial rhythm was non-shockable (pulseless electrical activity (PEA)/asystole) in 91% (*n* = 30); 94% (*n* = 31) had sustained return of spontaneous circulation (ROSC). The median time to ROSC was 3 (1–5) minutes. Patients with ICU-CA had significantly higher ICU mortality (61% vs. 37%). Multivariable logistic regression showed that the presence of ARDS (odds ratio (OR) 4.268, 95% confidence interval (CI) 1.211–15.036; *p* = 0.024) and high SAPS II (OR 1.031, 95% CI 0.997–1.065; *p* = 0.077) were independently associated with the occurrence of ICU-CA. A total of 18% of critically ill patients with COVID-19 suffered from a cardiac arrest within the intensive care unit. The occurrence of ICU-CA was associated with presence of ARDS and severity of illness.

## 1. Introduction

The severe acute respiratory syndrome coronavirus 2 (SARS-CoV-2) emerged in 2019 in China and caused a worldwide pandemic [1,2]. Although the majority of patients have an asymptomatic or mild course of disease, about 5% suffer from severe illness complicated by acute respiratory distress syndrome (ARDS) or other end-organ failure [3]. During the pandemic, an increase in out-of-hospital cardiac arrest (OHCA) cases was observed in different regions [4,5]. Relatively little is known about the occurrence of in-hospital cardiac arrest (IHCA) during the coronavirus disease 2019 (COVID-19) pandemic [6,7]. One study reported a shift of cardiac arrest (CA) characteristics during the pandemic [6]. However, different studies reported on characteristics of IHCA in patients with COVID-19 [8,9,10,11]. In most studies, a devastating mortality ranging from 88–100% has been reported [8,10,11,12]. A recent multicenter study from the US showed large variations in outcome after IHCA between centers [13]. A large number of IHCA in the recent literature occurred within the intensive care unit (ICU) [8,12,13]. Cardiac arrest in the ICU, also known as ICU-CA, represents a less investigated subgroup of IHCA. Due to clinically important differences, ICU-CA should be considered separately from IHCA in a general ward [14]. The incidence of ICU-CA varies greatly in the literature (4–78/1000 admissions) [14,15]. Survival rates to discharge after ICU-CA are diverse and range from 2 to 79%, mainly depending on the ICU population studied [14,15]. However, one recent study in critically ill patients with COVID-19 reported a mortality rate of 88% [11]. Mortality after CA is mainly triggered by post-CA shock and brain injury [16]. Further, it has been shown that organ failure before and after ICU-CA is common and the severity of illness after ICU-CA and the occurrence of liver failure are independent predictors of mortality [17]. To date, most studies focused on the characteristics of IHCA without comparing clincical characteristics to other patients with COVID-19. Further, studies focusing on critically ill patients and risk factors for ICU-CA are scarce. 

However, data on ICU-CA within critically ill patients with COVID-19 are limited. In the present study, we aimed to investigate the occurrence, risk factors and outcome of patients with COVID-19 suffering from ICU-CA. Further, we investigated factors associated with favorable outcome within ICU-CA.

## 2. Materials and Methods

### 2.1. Study Population, Design and Ethics

This was a retrospective analysis of data prospectively recorded at the University Medical Centre Hamburg-Eppendorf (Germany). All consecutive adult patients with COVID-19 admitted (27 February 2020–14 January 2021) to the Department of Intensive Care Medicine were included. The study complied with the Declaration of Helsinki. The Ethics Committee of the Hamburg Chamber of Physicians was informed about the study (No.: WF-60/21). The requirement for informed patient consent was waived due to the use of only anonymized data collected during routine clinical care. The last day of follow-up was 15 March 2021.

### 2.2. Inclusion and Exclusion Criteria

We included all consecutive critically ill adult patients (≥18 years) with confirmed COVID-19 who were admitted to the Department of Intensive Care Medicine during the study period. Patients <18 years or with a prior event of OHCA/IHCA event before ICU admission, as well as cardiac re-arrest after ICU admission, were not considered as ICU-CA and were therefore excluded. Patients without confirmed COVID-19 were excluded.

### 2.3. Study Definitions and Patient Management

Cardiac arrest was defined as cessation of circulation, and therefore, an indication for chest compression and/or cardiac defibrillation in patients who had a pulse and circulation at the time of ICU admission. The sustained return of spontaneous circulation (ROSC) was defined as stable circulation for at least 20 min. The assessment of neurological outcome was performed within routine clinical practice using cerebral performance categories (CPCs) after ICU-CA and during follow-up. A CPC score of 1–2 was defined as a favorable neurological outcome, and a score of 3–5 was defined as an unfavorable neurological outcome. Survival was assessed through the end of the ICU stay. Cardiopulmonary resuscitation and post-CA care were performed in accordance with the European Resuscitation Council guidelines [18,19]. Data were collected according to Utstein-style guidelines [20]. Hypoxic liver injury (HLI) was diagnosed according to established criteria [21]. Confirmed COVID-19 was defined as a positive result on a reverse transcriptase-polymerase chain reaction, and only laboratory-confirmed cases were counted as COVID-19. ARDS was defined using the PaO_2_/FiO_2_ ratio (Horowitz index) according to the Berlin definition [22,23]. The severity of illness was evaluated by the sequential organ failure assessment (SOFA) score [24] and simplified acute physiology (SAPS II) [25] score. The Charlson comorbidity index (CCI) [26] was calculated in all patients.

### 2.4. Data Collection

Data were collected through electronic patient data management systems (PDMS, Integrated Care Manager^®^ (ICM), Version 9.1—Draeger Medical, Luebeck, Germany; Soarian Clinicals, Version 4.3.200—Cerner Health Service, Inc. (North Kansas City, Missouri, USA) and consisted of age, sex, comorbidities, admission diagnosis, length of ICU stay, treatment modalities, organ support (mechanical ventilation, vasopressor, renal replacement therapy (RRT), blood transfusions, antibiotics, antivirals, etc.), laboratory parameters and further clinical parameters of interest through the end of ICU stay. Pre-existing medication was recorded based on known regular medications and medications on admission. Laboratory assessment was performed daily as part of the clinical routine.

### 2.5. Statistical Analysis

The results are presented as counts and relative frequencies or medians and 25–75% interquartile ranges (IQRs). Binary variables were compared via chi-square analysis/Fisher’s exact test, as appropriate. Metric variables were compared via the Mann–Whitney U test. We used multivariable logistic regression to investigate risk factors associated with occurrence of ICU-CA. Factors of clinical relevance were selected and included. A stepwise backward elimination approach that gradually reduces the initial model was used; variables that caused a change in estimates >10%/statistically significant remained in the model. Statistical analysis was conducted using IBM SPSS Statistics Version 24.0 (IBM Corp., Armonk, NY, USA). The study was prepared in accordance with the STROBE (STrengthening the Reporting of OBservational studies in Epidemiology) recommendations.

## 3. Results

### 3.1. Study Population

**Figure 1:**
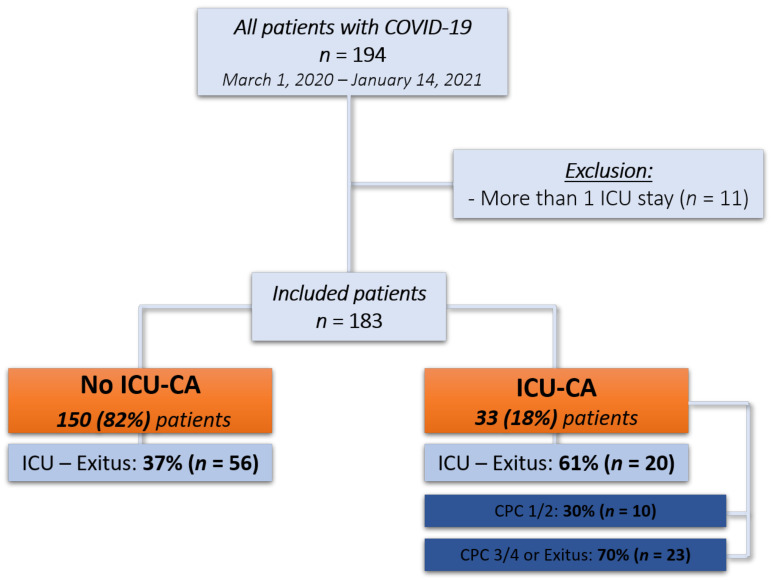
Study flow chart.

During the study period (1 March 2020 to 14 January 2021), a total of 194 patients with confirmed COVID-19 were treated at the Department of Intensive Care Medicine at the University Medical Centre Hamburg-Eppendorf. After the exclusion of cases who were admitted more than one time in the aforementioned time frame, we could include 183 patients in the final cohort. Within the cohort, we could identify 33 (18%) patients who had suffered from an ICU-CA and 150 (82%) patients who did not suffer from an ICU-CA (see study flow chart, Figure 1). 

### 3.2. Baseline and ICU Characteristics of the Study Populations

Detailed baseline characteristics are shown in Table 1. The median age of the study population was 63 (55–73) years and 66% (*n* = 120) were male. Comorbidities, displayed by CCI, were a median of 2 (1–3) points. Leading comorbidities were arterial hypertension (57%, *n* = 105), diabetes mellitus (33%, *n* = 60), malignant condition (tumor or hematologic malignancy) (25%, *n* = 45), coronary heart disease (19%, *n* = 34), chronic kidney disease (15%, *n* = 28) and chronic respiratory disease (15%, *n* = 27). Demographic characteristics and comorbidities did not differ significantly between both groups (see Table 1 and Supplementary Table S1). The time from the first positive test for COVID-19 to ICU admission was a median of 5 (1–12) days and differed between both groups (ICU-CA: 8 days vs. no ICU-CA: 5 days; *p* = 0.032). Presenting COVID-19 symptoms did not differ significantly between both groups. 

Disease severity displayed by SAPS II (median: 44 points vs. 39 points) and SOFA score (median: 12 points vs. 7 points) on admission was significantly higher in patients with ICU-CA. Non-invasive ventilation (NIV) and high flow nasal cannula (HFNC) was comparably used in both groups. Mechanical ventilation was necessary in 73% (*n* = 133) of the whole cohort and was significantly more common in patients with ICU-CA (97% vs. 67%). ARDS was present in 68% (*n* = 124) of patients, in 91% (*n* = 30) with and in 63% (*n* = 94) without ICU-CA (*p* = 0.002). ARDS management included prone positioning (*n* = 95), neuromuscular blockade (*n* = 42), inhaled nitric oxide (*n* = 57) and glucocorticoid therapy (*n* = 118) within the whole cohort and were evenly balanced between both groups. Due to severe ARDS accompanied by life-threatening hypoxemia, veno-venous extracorporeal membrane oxygenation (ECMO) was established in 28% (*n* = 52) and was significantly more common in patients with ICU-CA (52% vs. 23%). The use of renal replacement therapy (RRT) was frequent in both groups (67% vs. 43%, *p* = 0.015). Further, patients with ICU-CA had a median longer ICU stay, with 21 (8–32) compared to 12 (5–24) days.

### 3.3. Characteristics of Intensive Care Unit Cardiac Arrest

The characteristics of ICU-CA are shown in detail in Table 2 and Supplementary Table S2. Thirty-three (18%) patients of the cohort suffered from cardiac arrest during the ICU stay. The median stay before ICU-CA was 6 (1–17) days. A total of 33% (*n* = 11) of ICU-CAs occurred during the first 24 h of ICU stay. The initial cardiac rhythm was non-shockable (PEA/asystole) in 91% (*n* = 30). Defibrillation was performed in 15% (*n* = 5) of the patients. A total of 94% (*n* = 31) had sustained ROSC, and 21% (*n* = 7) suffered from cardiac re-arrest. The median time to ROSC was 3 (1–5) minutes. A mechanical compression device was used in one patient with prolonged CPR. The cause of cardiac arrest was presumed non-cardiac in 85% (*n* = 28). Epinephrine was used during CA in 79% (*n* = 26); the median epinephrine dose was 1 (1–2) mg. Targeted temperature management was applied in 48% (*n* = 16). The highest lactate was 4.6 (3.1–8.3) mmol/l and the lowest pH was 7.2 (7.12–7.3), after ICU-CA, respectively.

Before cardiac arrest, the SAPS II and SOFA score were median 44 (37–52) and 12 (10–15) points, respectively. During the ICU stay 97% (*n* = 32) patients with ICU-CA received MV. Prior to ICU-CA 27% (*n* = 9) received NIV/HFNC and 70% (*n* = 23) were mechanically ventilated. The median *p*/F-Ratio before CA was 114 (80–154). Overall, 53% (*n* = 17) had support via ECMO. Ten (30%) patients had veno-venous ECMO before ICU-CA, and 8 (24%) patients were on ECMO at the time of ICU-CA. One patient received E-CPR due to refractory cardiac arrest. Peri-arrest ECMO was established in 15% (*n* = 5) due to persistent hypoxemia; all were configured as veno-venous ECMO. A total of 97% (*n* = 32) of patients received vasopressor therapy during the ICU stay, and 82% (*n* = 27) were on vasopressors before CA (for detailed hemodynamic characteristics, see Supplementary Table S3). Of interest, echocardiography assessment revealed that three (9%) patients presented cor pulmonale. A total of 24% (*n* = 8) received RRT before CA, and RRT was newly initiated in 42% (*n* = 14) after CA. Liver dysfunction was observed frequently: 21% (*n* = 7) suffered from hypoxic liver injury and 45% (*n* = 15) from cholestasis. 

### 3.4. Survival, Functional Outcome and Risk Factors for ICU-CA

Of the 183 included patients, 42% (*n* = 76) did not survive the ICU stay. Patients with ICU-CA had a significant higher ICU mortality (61%, *n* = 20) as compared to other patients (37%, *n* = 56). Of patients with ICU-CA, 24% (*n* = 8) died within ICU-CA 24 h after CA. At ICU discharge, 30% (*n* = 10) of patients with ICU-CA presented with favorable neurological outcome (CPC 1/2), and 70% (*n* = 23) with unfavorable (CPC 3/4 or death). Multivariable logistic regression analysis revealed that the most pertinent factors associated with the occurrence of ICU-CA were the presence of ARDS (OR 4.268, 95% CI (1.211–15.036); *p* = 0.024) and high SAPS II (OR 1.031, 95% CI (0.997–1.065); *p* = 0.077) (see Supplementary Table S4).

### 3.5. Factors Associated with Unfavorable Outcome in Patients with ICU-CA

Detailed characteristics on ICU-CA with favorable and unfavorable outcome are shown in Table 2 and Supplementary Table S2. Cardiac arrest characteristics were comparable between both groups. A longer total resuscitation time (median 4 vs. 2 min) and a higher rate of cardiac re-arrest (30% vs. 0%, *p* = 0.049) was observed in patients with unfavorable outcome. Lactate (6.1 vs. 3.4 mmol/L) was higher and pH levels (7.2 vs. 7.4) lower after CA in patients with unfavorable outcome (both *p* < 0.05). The SOFA score before and after ICU-CA was significantly higher in patients with unfavorable outcome. Interventions in place before ICU-CA were well balanced between both groups. Vasopressor therapy was in place in 96% with unfavorable outcome, as compared to 50% with favorable outcome (*p* = 0.002). ARDS management, including prone positioning, neuromuscular blockage, corticosteroids and inhaled vasodilatory treatment, was comparable in both groups. Complications during ICU stay were comparable between both groups, and the occurrence of cholestasis was significantly higher in patients with ICU-CA and unfavorable outcome (61% vs. 10%). 

## 4. Discussion

We investigated the occurrence, characteristics, and outcome as well as risk factors for ICU-CA in critically ill patients with COVID-19. We found that ICU-CA occurred in 18% of critically ill patients with COVID-19 and was related to more advanced stages of ARDS and severity of illness. To our knowledge, this is the most comprehensive study focusing on factors for occurrence of ICU-CA in patients with COVID-19. Furthermore, this is the first study showing detailed post-ICU-CA characteristics in patients with COVID-19. 

The reported incidence of ICU-CA in the literature varies greatly, ranging from 4—78/1000 ICU admissions [14,15,27]. However, the incidence declined in past years, probably an expression of advances in the management and treatment of critically ill patients [14]. The highest incidence rates were reported in patients with underlying malignant conditions within a comprehensive cancer center [28]. Mainly, the large variation in incidence is explained by the setting and the cohort studied. Two recent studies in a mixed ICU cohort showed an incidence of 22–23/1000 ICU admissions [17,29]. To our knowledge, only one previous study focused on the occurrence and incidence of cardiac arrest in critically ill patients with COVID-19 treated in the intensive care unit [11]. The study by Hayek and colleagues proposed an incidence of about 80/1000 ICU admissions, whereas we observed an incidence of 180/1000 ICU admissions in the present study. This large difference can potentially be the consequence of different factors. In direct comparison, the median SOFA score on admission was considerably higher in our cohort. Almost three-fourths of patients were mechanically ventilated in our study, which is comparable to the study by Hayek and colleagues [11]. However, only 4% were treated by ECMO as compared to 28% in our cohort, which reflects a considerably higher severity of illness. This would be in line with our finding that the severity of ARDS is the strongest risk factor for the occurrence of ICU-CA accompanied by the high incidence observed. However, whether this finding is unique in patients with COVID-19 has to be further elucidated. Earlier studies in the setting of ICU-CA did not focus on patients with respiratory insufficiency or ARDS and were mainly conducted in mixed ICU settings. Furthermore, our hospital acts as a referral center specialized in the care of critically ill patients with ARDS. Therefore, we are treating patients who probably have a more severe course of disease and our findings may not be transferable to other hospitals in different settings. 

Critically ill patients with COVID-19 mainly suffer from respiratory insufficiency and many patients require mechanical ventilation. Patients with a need for oxygen support are at high risk for rapidly worsening and the development of respiratory failure. Of interest, the deterioration of SpO_2_ in combination with higher early warning scores have recently been described as predictors for worsening in patients with COVID-19 [30,31]. Therefore, close monitoring and early transfer to a more staffed and monitored setting seems reasonable. However, we strictly followed a protocol for early ICU admission of deteriorating patients with COVID-19 in our institution. We observed a high rate of non-shockable rhythm (91%), mainly owing to a non-cardiac cause, which is in line with earlier reports of cardiac arrest in COVID-19 [6,10,11,32]. We also observed a high rate of severe ARDS in our cohort, highlighted by the fact that 24% of patients had an VV-ECMO in place at the time of ICU-CA. The observed resuscitation times in our cohort were mainly short and sustained ROSC was achieved in 94% of patients. The resuscitation times were slightly longer as compared to an earlier study in the mixed ICU, which may be explained by the need for personal protective equipment and the potential delay in the initiation of CPR. However, compared to other studies on COVID-19, we observed quite low resuscitations times [11]. To date, reported outcomes after IHCA/ICU-CA in patients with COVID-19 are worse, ranging from 88–100%, and have led to discussions about futility and limitation of care [8,10,11,12,32]. Although we observed high mortality in our cohort, it was substantially lower than previously reported. We observed mainly similar cardiac arrest characteristics, but different factors can contribute to better outcome. Firstly, all patients suffered from CA in the ICU. Due to higher nurse/doctor staffing and continuous monitoring, this could explain faster response times and lower times to ROSC. Secondly, some reports derived from regions with a high excessive patient load during the pandemic, possibly contributing to factors associated with worse outcome. 

Organ dysfunction and organ failure after CA is frequently observed [16,33,34,35,36,37]. The high morbidity and mortality after CA were shown to be mainly triggered by post-CA shock and brain injury [16,38]. The effects of pre-existing organ dysfunction and organ support are less clear. In our cohort, more than 90% of patients had invasive or non-invasive respiratory support at the time of ICU-CA. One large study observed that mechanical ventilation at the time of CA is associated with noticeably decreased survival [39]. However, we did not observe differences regarding survival in patients with ICU-CA. About 80% of patients had vasopressor support in place at the time of ICU-CA; we observed an association with an unfavorable outcome. This is in line with two previous studies which also found an association with the pre-arrest use of vasopressors with unfavorable outcome [40,41]. About one-fourth of the patients received RRT prior to ICU-CA and RRT was newly initiated in 42% of patients after ICU-CA. We did not observe an association with unfavorable outcome as compared to earlier studies in OHCA patients [34]. Of interest, we observed that 21% suffered from hypoxic liver injury and 45% from cholestasis, which is in line with earlier reports in critically ill patients with COVID-19 [42]. Although hypoxic liver injury was shown to be associated with unfavorable outcome in ICU-CA, we did not observe this in the present study [17,33]. However, cholestasis was significantly associated with unfavorable outcome in our cohort. This was previously shown in critically ill patients, but not in patients after CA [43].

We observed a significantly higher severity of illness in patients suffering from ICU-CA compared to patients without ICU-CA. Of interest, these differences were already evident on admission, represented by SOFA and SAPS II, and we found that a high SAPS II score was associated with the occurrence of ICU-CA. Although the median time from admission to ICU-CA was 6 days, about one-third of the cohort suffered ICU-CA within 24 h of ICU admission, highlighting this quite vulnerable patient group. This is also comparable to earlier studies on ICU-CA in mixed ICUs [17]. In our cohort, we observed that severity of illness (SOFA) before and after CA was significantly associated with unfavorable outcome. To our knowledge, only the APACHE III score was evaluated for outcome prediction in ICU-CA before and showed moderate predictive performance [44]. However, SOFA score can be easily and quickly assessed. Future studies have to clarify if there is a value of SOFA score in outcome prediction compared to other scoring systems used after ICU-CA. 

This study has several limitations. Firstly, we present data deriving from a single center very experienced in the management of CA and post-CA care as well as ARDS and COVID-19. Thus, our results may not be transferrable to other settings. Secondly, we cannot entirely exclude the possibility that changes in general management and advances in critical care of patients with COVID-19 had influence on the occurrence and outcome of ICU-CA. Thirdly, the data were collected retrospectively from a prospectively documented PDMS filled by trained ICU staff. Fourthly, residual confounding is a matter of concern and cannot be entirely excluded. Fifthly, our cohort of patients with ICU-CA was small. Future studies have to clarify and confirm our findings in a larger setting. 

## 5. Conclusions

In conclusion, this is the first study in critically ill patients with COVID-19 focusing on the post-CA course of ICU-CA. We found that 18% of critically ill patients suffered from ICU-CA with a corresponding incidence of 180/1000 ICU admissions. The occurrence of ICU-CA was strongly associated with higher stages of ARDS and severity of illness on admission. Although patients with ICU-CA suffered from high mortality, cardiopulmonary resuscitation in this selected patient cohort is not futile.

## Figures and Tables

**Table 1:** Baseline and ICU-characteristics of patients stratified according to ICU cardiac arrest (ICU-CA) and no ICU cardiac arrest (no ICU-CA).

Parameters	*All Patients*	*ICU-CA*	*No ICU-CA*	*p-Value*
	*(n = 183)*	*(n = 33)*	*(n = 150)*	
**Demographics**				
Age, years *median (IQR)*	63 (55–73)	64 (55–75)	62 (55–73)	0.627
Gender, male *n (%)*	120 (66)	20 (61)	100 (67)	0.507
Height, cm *median (IQR)*	175 (168–180)	172 (166–180)	175 (169–180)	0.238
Weight, kg *median (IQR)*	85 (73–100)	85 (72–100)	84 (73–100)	0.947
BMI, kg/m^2^ *median (IQR)*	27 (24–32)	29 (24–33)	27 (24–32)	0.398
**Comorbidities**				
Charlson comorbidity index, pts.; *median (IQR)*	2 (1–3)	2 (1–3)	2 (1–3)	0.801
Arterial hypertension, *n (%)*	105 (57)	22 (67)	83 (55)	0.233
Coronary heart disease, *n (%)*	34 (19)	7 (21)	27 (18)	0.534
Chronic kidney disease, *n (%)*	28 (15)	3 (9)	25 (17)	0.274
Chronic respiratory disease, *n (%)*	27 (15)	5 (15)	22 (15)	0.943
Diabetes, *n (%)*	60 (33)	12 (36)	48 (32)	0.259
Malignant condition, *n (%)*	45 (25)	9 (27)	36 (24)	0.693
**COVID-19**				
Positive test to ICU, days *median (IQR)*	5 (1–12)	8 (3–17)	5 (1–11)	0.032
Cough, *n (%)*	82 (44)	16 (48)	66 (44)	0.613
Shortness of breath, *n (%)*	111 (61)	19 (58)	92 (61)	0.689
Fever, *n (%)*	81 (44)	13 (39)	68 (45)	0.534
Fatigue, *n (%)*	24 (13)	4 (12)	20 (13)	0.852
Myalgia, *n (%)*	9 (5)	2 (6)	7 (5)	0.737
**Disease Severity on admission**				
SAPS II (pts.) *median (IQR)*	40 (33–48)	44 (37–52)	39 (32–45)	0.016
SOFA (pts.) *median (IQR)*	7 (3–12)	12 (6–13)	7 (3–11)	0.004
**ICU Procedures**				
Mechanical ventilation, *n (%)*	133 (73)	32 (97)	101 (67)	0.001
HFNC, *n (%)*	67 (37)	8 (24)	59 (39)	0.103
NIV, *n (%)*	49 (27)	9 (27)	40 (27)	0.943
ECMO, *n (%)*	52 (28)	17 (52)	35 (23)	0.001
Vasopressor, *n (%)*	145 (79)	32 (97)	113 (75)	0.006
RRT, *n (%)*	87 (48)	22 (67)	65 (43)	0.015
**ARDS and Management**				
ARDS	124 (68)	30 (91)	94 (63)	0.002
-Mild	7 (4)	1(3)	6 (4)	0.314
-Moderate	24 (13)	2 (6)	22 (15)	0.037
-Severe	93 (51)	27(82)	66 (44)	0.049
Inhaled vasodilator	57 (31)	15 (45)	42 (28)	0.05
Prone positioning	95 (52)	17 (52)	78 (52)	0.96
Neuromuscular blockade	42 (23)	9 (27)	33 (22)	0.514
Steroid therapy	118 (64)	23 (70)	95 (63)	0.489
**Complications**				
Heart failure, *n (%)*	8 (4)	2 (6)	6 (4)	0.6
Pulmonary embolism, *n (%)*	13 (7)	2 (6)	11 (7)	0.797
Deep vein thrombosis, *n (%)*	15 (8)	1 (3)	14 (9)	0.232
Myocardial infarction, *n (%)*	7 (4)	3 (9)	4 (3)	0.082
Septic shock, *n (%)*	80 (44)	20 (61)	60 (40)	0.035
**Outcome**				
ICU mortality, *n (%)*	76 (42)	20 (61)	56 (37)	0.014
In-hospital mortality, *n (%)*	78 (43)	20 (61)	58 (39)	0.021
Length of stay—ICU, days *median (IQR)*	13 (5–25)	21 (8–32)	12 (5–24)	0.159

Abbreviations: cm, centimeter; BMI, body mass index; kg, kilogram; ICU, intensive care unit; IQR, interquartile range; *n*, number; pts, points; min, minute; MAP, mean arterial pressure; COVID-19, coronavirus disease 2019; HFNC, high flow nasal cannula; NIV, non-invasive ventilation; RRT, renal replacement therapy; ECMO, extracorporeal membrane oxygenation.

**Table 2:** Cardiac arrest and ICU characteristics of patients with ICU-CA stratified according favorable and unfavorable outcome.

Parameters	*All Patients*	*ICU-CA Favorable*	*ICU-CA Unfavorable*	*p-Value*
	*(n = 33)*	*(n = 10)*	*(n = 23)*	
**Cardiac arrest Characteristics**				
Initial rhythm shockable (VT/VF), *n (%)*	3 (9)	2 (20)	1 (4)	0.151
Defibrillation, *n (%)*	5 (15)	1 (10)	4 (17)	0.586
Sustained ROSC, *n (%)*	31 (94)	10 (100)	21 (91)	0.336
Cardiac re-arrest, *n (%)*	7 (21)	0 (0)	7 (30)	0.049
Presumed non-cardiac cause, *n (%)*	28 (85)	8 (80)	20 (87)	0.609
Epinephrine *median (IQR)*	1 (1–2)	2 (1–2)	1 (1–2.3)	0.501
*Ischemic time, min; median (IQR)*				
-No-flow	0 (0–0)	0 (0–0)	0 (0–0)	0.363
-Total resuscitation time	3 (1–5)	2 (0.8–4.5)	4 (1–6)	0.354
Targeted temperature management, *n (%)*	16 (48)	3 (30)	13 (57)	0.161
Use of mechanical compression system, *n (%)*	1 (3)	0 (0)	1 (4)	0.697
E-CPR, *n (%)*	1 (3)	0 (0)	1 (4)	0.503
VV-ECMO—before CA, *n (%)*	8 (24)	3 (30)	5 (22)	0.611
**ICU characteristics**				
*Severity of illness*				
SAPS II (pts.) *median (IQR)*	44 (37–52)	40.5 (35–53)	47 (38–52)	0.472
SOFA—before CA (pts.) *median (IQR)*	12 (10–15)	9 (6–12)	13 (12–15.5)	0.038
SOFA—after CA (pts.) *median (IQR)*	15 (12–16)	12 (9–13)	16 (13.5–17)	0.01
SOFA—24 h after CA (pts.) *median (IQR)*	14 (10–17)	8 (7–13)	16 (13.5–17)	0.002
***Lab values—post CA** median (IQR)*				
Lactate—highest after CA, mmol/l	4.6 (3.1–8.3)	3.4 (1.4–4.5)	6.1 (4.2–12.7)	0.016
pH—lowest after CA	7.2 (7.12–7.3)	7.4 (7.18–7.46)	7.2 (7.06–7.25)	0.034
Horowitz Index *median (IQR)*	114 (80–154)	93 (65–174)	97 (67–140)	0.685
*Procedures/complications during ICU*				
Mechanical ventilation, *n (%)*	32 (97)	10 (100)	22 (96)	0.503
Extracorporeal membrane oxygenation, *n (%)*	17 (53)	6 (60)	11 (48)	0.52
Vasopressor therapy, *n (%)*	32 (97)	9 (90)	23 (100)	0.503
Renal replacement therapy, *n (%)*	22 (67)	5 (50)	17 (74)	0.181
Coronary angiography—post CA, *n (%)*	1 (3)	0 (0)	1 (4)	0.891
Hypoxic liver injury, *n (%)*	7 (21)	2 (20)	5 (22)	0.911
Cholestasis–Bilirubin >2 mg/dl, *n (%)*	15 (45)	1 (10)	14 (61)	0.007

Abbreviations: CA, cardiac arrest; E-CPR, extracorporeal cardiopulmonary resuscitation; ICU, intensive care unit; IQR, inter quartile range; *n*, number; min, minute; pts., points; ROSC, return of spontaneous circulation; SAPS, Simplified Acute Physiology Score; SOFA, Sequential Organ Failure Assessment; VF, ventricular fibrillation; VT, ventricular tachycardia; MAP, mean arterial pressure.

## Data Availability

Data sharing is not applicable to this article.

## Supplementary Materials

The following are available online at https://www.mdpi.com/article/10.3390/jcm10102195/s1, Table S1—pre-existing comorbidities of patients stratified according to ICU cardiac arrest (ICU-CA) and no ICU cardiac arrest (No ICU-CA); Table S2—detailed characteristics of respiratory support and ICU therapies in patients with ICU-CA stratified according favorable and unfavorable outcome; Table S3—hemodynamic characteristics of in patients with ICU-CA stratified according favorable and unfavorable outcome; Table S4—logistic regression model for factors associated with occurrence of ICU-CA.
