# Supplementary material for: Characteristics and Risk Factors for Intensive Care Unit Cardiac Arrest in Critically Ill Patients with COVID-19—A Retrospective Study
Source: J Clin Med. 2021 May 19;10(10):2195. doi: 10.3390/jcm10102195 (PMC8160993; doi:10.3390/jcm10102195)
Supplement: Supplementary file 1 [file jcm-10-02195-s001.zip › jcm-1215208-supplementary.pdf]

Supplementary Table S1 – Pre-existing comorbidities of patients stratified according to ICU cardiac arrest (ICU-CA) and no ICU cardiac arrest (No ICU-CA)

Parameters	***All patients*** *(n=183)*	***ICU-CA*** *(n=33)*	***No ICU-CA*** *(n=150)*	*p-value*
**AIDS** *n (%)*	0 (0)	0 (0)	0 (0)	1
**Cerebral arterial disease** *n (%)*	32 (17)	4 (12)	28 (19)	0.370
**Chronic lung disease** *n (%)*	27 (15)	5 (15)	22 (15)	0.943
**Chronic kidney disease** *n (%)*	28 (15)	3 (9)	25 (17)	0.274
**Congestive heart disease** *n (%)*	26 (14)	5 (15)	21 (14)	0.864
**Connective tissue disease** *n (%)*	12 (7)	1 (3)	11 (7)	0.366
**Coronary heart disease** *n (%)*	34 (19)	7 (21)	27 (18)	0.534
**Dementia** *n (%)*	3 (2)	0 (0)	3 (2)	0.951
**Diabetes mellitus** *n (%)*	60 (33)	12 (36)	48 (32)	0.259
**Liver cirrhosis** *n (%)*	4 (2)	0 (0)	4 (3)	0.413
**Peripheral arterial disease** *n (%)*	9 (5)	3 (9)	6 (4)	0.221
**Malignancy (Tumour)** *n (%)*	24 (13)	5 (15)	19 (13)	0.808

Abbreviations: AIDS, acquired immune deficiency syndrome; n, number;

Supplementary Table S2 – Detailed characteristics of respiratory support and ICU therapies in patients with ICU-CA stratified according favorable and unfavorable outcome

Parameters	***All patients*** *(n=33)*	***ICU-CA favorable*** *(n=10)*	***ICU-CA non-favorable*** *(n=23)*	*p-value*
**Characteristics of respiratory failure**				
**Respiratory support (before CA)**				
- Non-invasive ventilation *n (%)*	9 (27)	4 (40)	5 (22)	0.279
- Mechanical ventilation *n (%)*	23 (70)	6 (60)	17 (74)	0.424
- ECMO in place *n (%)*	8 (24)	3 (30)	5 (22)	0.611
**P/F Ratio**				
- Worst P/F *median (IQR)*	56 (46 – 83)	58 (55 – 107)	85 (58 – 116)	0.703
- P/F before CA *median (IQR)*	114 (80 – 154)	167 (78 – 298)	111 (81 – 137)	0.251
- P/F after CA *median (IQR)*	114 (80 – 154)	93 (65 – 174)	97 (67 – 140)	0.685
- P/F 24h after CA *median (IQR)*	108 (82 – 206)	202 (140 – 258)	92 (80 – 119)	0.019
**ARDS Management**				
- Prone Positioning *n (%)*	17 (52)	5 (50)	12 (52)	0.909
- Neuromuscular Blockage *n (%)*	9 (27)	3 (30)	6 (26)	0.817
- Corticosteroids *n (%)*	23 (70)	6 (60)	17 (74)	0.424
- Inhaled Vasodilators *n (%)*	15 (45)	4 (30)	11 (48)	0.678
**Extracorporeal membrane oxygenation**				
**-** ECMO overall *n (%)*	17 (52)	6 (60)	11 (48)	0.520
- ECMO before CA *n (%)*	10 (30)	5 (50)	5 (22)	0.104
- E-CPR *n (%)*	1 (3)	0 (0)	1 (4)	0.503
- Peri-arrest ECMO *n (%)*	5 (15)	1 (10)	4 (17)	0.586
ICU Intervention - in place before CA				
Mechanical ventilation *n (%)*	23 (70)	6 (60)	17 (74)	0.424
Non-invasive mechanical ventilation/HFNC	9 (27)	4 (40)	5 (22)	0.279
n (%)	8 (24)	3 (30)	5 (22)	0.611
Renal replacement therapy *n (%)*	14 (42)	2 (20)	12 (52)	0.341
Renal replacement therapy – new after ICU-CA	27 (82)	5 (50)	22 (96)	0.002
Vasopressor therapy *n (%)*				

Abbreviations: CA, cardiac arrest; cm, centimeter; E-CPR, extracorporeal cardiopulmonary resuscitation; ECMO, extracorporeal membrane oxygenation; ICU, intensive care unit; IQR, inter quartile range; n, number; pts., points; MAP, mean arterial pressure;

Supplementary Table S3 – Hemodynamic characteristics of in patients with ICU-CA stratified according favorable and unfavorable outcome

Parameters	***All patients*** *(n=33)*	***ICU-CA favorable*** *(n=10)*	***ICU-CA non-favorable*** *(n=23)*	*p-value*
**Hemodynamic characteristics**				
Hemodynamic parameters - before CA				
Heart rate *median (IQR)*	100 (85 – 118)	107 (91 – 113)	100 (85 – 120)	0.540
Mean arterial pressure *median (IQR)*	66 (58 – 77)	77 (61 – 84)	65 (58 – 75)	0.171
Vasopressor therapy *n (%)*	27 (82)	5 (50)	22 (96)	0.002
Norepinephrine dose* *median (IQR)*	0.397 (0.130 – 0.779)	0.117 (0.081 – 0.149)	0.535 (0.216 – 0.895)	0.068
Hemodynamic parameters – post CA				
Heart Rate – after CA *median (IQR)*	123 (104 – 138)	116 (82 – 128)	123 (114 – 142)	0.150
MAP – after CA *median (IQR)*	73 (66 – 95)	71 (69 – 85)	73 (66 – 97)	0.966
Vasopressor therapy *n (%)*	31 (94)	8 (80)	23 (100)	0.027
Norepinephrine dose* *median (IQR)*	0.691 (0.168 – 1.253)	0.158 (0.120 – 0.255)	0.879 (0.329 – 1.558)	0.005
Hemodynamic parameters – 24h after CA				
Heart rate *median (IQR)*	83 (75 – 111)	77 (73 – 89)	86 (75 – 113)	0.541
Mean arterial pressure *median (IQR)*	74 (67 – 85)	74 (69 – 76)	74 (66 – 86)	0.890
Vasopressor therapy *n (%)*	24 (77)	8 (80)	16 (70)	0.063
Norepinephrine dose* *median (IQR)*	0.175 (0.103 – 0.367)	0.070 (0.033 – 0.137)	0.259 (0.163 – 0.448)	0.005

Abbreviations: CA, cardiac arrest; IQR, inter quartile range; n, number; MAP, mean arterial pressure; * µg/kg/min

Supplementary Table S4 – Logistic regression model for factors associated with occurrence ICU-CA

	Covariables	OR (95% CI)	*p* value
Step 1:	Age (years)	1.004 (0.969 to 1.040)	0.819
	SAPS II admission (points)	1.022 (0.981 to 1.065)	0.302
	Vasopressor (yes vs no)	2.535 (0.223 to 28.827)	0.453
	Renal replacement therapy (yes vs no)	1.541 (0.514 to 4.619)	0.440
	Septic shock (yes vs no)	0.822 (0.264 to 2.558)	0.736
	ARDS (yes vs no)	2.590 (0.543 to 12.352)	0.233
	SOFA admission (points)	1.018 (0.918 to 1.128)	0.739
Step 2:	SAPS II admission (points)	1.024 (0.985 to 1.064)	0.226
	Vasopressor (yes vs no)	2.606 (0.232 to 29.232)	0.438
	Renal replacement therapy (yes vs no)	1.542 (0.514 to 4.625)	0.439
	Septic shock (yes vs no)	0.812 (0.262 to 2.513)	0.718
	ARDS (yes vs no)	2.573 (0.540 to 12.263)	0.235
	SOFA admission (points)	1.014 (0.920 to 1.118)	0.781
Step 3:	SAPS II admission (points)	1.026 (0.990 to 1.063)	0.164
	Vasopressor (yes vs no)	2.722 (0.248 to 29.904)	0.413
	Renal replacement therapy (yes vs no)	1.528 (0.510 to 4.576)	0.449
	Septic shock (yes vs no)	0.828 (0.270 to 2.538)	0.741
	ARDS (yes vs no)	2.638 (0.561 to 12.417)	0.220
Step 4:	SAPS II admission (points)	1.025 (0.989 to 1.061)	0.175
	Vasopressor (yes vs no)	2.785 (0.255 to 30.418)	0.401
	Renal replacement therapy (yes vs no)	1.380 (0.552 to 3.453)	0.491
	ARDS (yes vs no)	2.466 (0.552 to 11.019)	0.237
Step 5:	SAPS II admission (points)	1.028 (0.994 to 1.063)	0.103
	Vasopressor (yes vs no)	2.846 (0.259 to 31.337)	0.393
	ARDS (yes vs no)	2.768 (0.638 to 12.006)	0.174
Final Model:	SAPS II admission (points)	1.031 (0.997 to 1.065)	0.077
	ARDS (yes vs no)	4.268 (1.211 to 15.036)	0.024

Abbreviations: ARDS, Acute Respiratory Distress Syndrome; CI confidence interval; HR, multivariable adjusted hazard ratio; SAPS II, Simplified Acute Physiology Score; SOFA**,** Sequential Organ Failure Assessment;
